# Comment on: ‘The swim-and-sink behaviour of copepods: a revisit to mechanical power requirement and a new hypothesis on function' Jiang (2023)

**DOI:** 10.1098/rsos.231180

**Published:** 2023-10-04

**Authors:** Daniel Weihs

**Affiliations:** Department of Aerospace, Technion, Haifa, Israel

**Keywords:** swim, sink, behaviour, copepods, mechanical

A recent paper by Jiang [1] reviews the swim-and-sink (better known as hop and sink) behaviour of small invertebrates such as copepods. The main point in the paper above was to show that our 50 year old suggestion [2, p. 802], that, to quote ‘alternating periods of active propulsion and passive gliding may be a common behaviour used in many diverse animals to conserve energy while maintaining a position against the force of gravity' is not correct for the copepods studied by Jiang.

This conclusion would be acceptable if the analysis provided, which now is based on computational fluid dynamics (CFD), is itself, more accurate. Unfortunately, even CFD solutions can only be as accurate as the assumptions used as inputs, and when those are inaccurate, so is the result. In Jiang [1] constant speeds, and their ratio, are assumed for both the swim, and the sink phases of motion. This is obviously impossible as this requires infinite accelerations and decelerations at the turning points.

The drag on animals of this size is highly speed dependent, as they move in a low Reynolds number transitional regime, so that the total force is not represented accurately anyway in a calculation assuming constant speeds, even when using averages. In reality, a sinusoidal variation of speed is probably a better approximation. The constant speed simplifying assumption cannot show the actual forces (and associated energies) accurately enough to shift the final conclusion from swim-and-sink saving energy, to costing extra energy.

So, actually, it is still unclear, if hop and sink behaviour saves or wastes energy relative to continuous hovering in copepods. I hope that this can be determined definitively in future work, now that the capabilities of CFD allow this.

## Data Availability

This article has no additional data.
